# Elucidating the Link Between Anxiety/Depression and Alzheimer’s Dementia in the Australian Imaging Biomarkers and Lifestyle (AIBL) Study

**DOI:** 10.1007/s44197-024-00266-w

**Published:** 2024-06-19

**Authors:** Liwei Ma, Edwin C. K. Tan, Ashley I. Bush, Colin L. Masters, Benjamin Goudey, Liang Jin, Yijun Pan, AIBL Research Group

**Affiliations:** 1 The Florey Institute of Neuroscience and Mental Health, Melbourne, Australia Victoria 3052; 2 Florey Department of Neuroscience and Mental Health, The University of Melbourne, Melbourne, Victoria 3052 Australia; 3 Faculty of Medicine and Health, The University of Sydney School of Pharmacy, The University of Sydney, Camperdown, New South Wales 2050 Australia; 4 Department of Organ Anatomy, Graduate School of Medicine, Tohoku University, Sendai, 980-8575 Miyagi Japan

**Keywords:** Alzheimer’s dementia, Anxiety, Cross-sectional analysis, Depression, Mild cognitive impairment, Longitudinal analysis

## Abstract

**Background:**

The associations between mood disorders (anxiety and depression) and mild cognitive impairment (MCI) or Alzheimer’s dementia (AD) remain unclear.

**Methods:**

Data from the Australian Imaging, Biomarker & Lifestyle (AIBL) study were subjected to logistic regression to determine both cross-sectional and longitudinal associations between anxiety/depression and MCI/AD. Effect modification by selected covariates was analysed using the likelihood ratio test.

**Results:**

Cross-sectional analysis was performed to explore the association between anxiety/depression and MCI/AD among 2,209 participants with a mean [SD] age of 72.3 [7.4] years, of whom 55.4% were female. After adjusting for confounding variables, we found a significant increase in the odds of AD among participants with two mood disorders (anxiety: OR 1.65 [95% CI 1.04–2.60]; depression: OR 1.73 [1.12–2.69]). Longitudinal analysis was conducted to explore the target associations among 1,379 participants with a mean age of 71.2 [6.6] years, of whom 56.3% were female. During a mean follow-up of 5.0 [4.2] years, 163 participants who developed MCI/AD (refer to as PRO) were identified. Only anxiety was associated with higher odds of PRO after adjusting for covariates (OR 1.56 [1.03–2.39]). However, after additional adjustment for depression, the association became insignificant. Additionally, age, sex, and marital status were identified as effect modifiers for the target associations.

**Conclusion:**

Our study provides supportive evidence that anxiety and depression impact on the evolution of MCI/AD, which provides valuable epidemiological insights that can inform clinical practice, guiding clinicians in offering targeted dementia prevention and surveillance programs to the at-risk populations.

**Supplementary Information:**

The online version contains supplementary material available at 10.1007/s44197-024-00266-w.

## Introduction

Alzheimer’s disease (AD) is a progressive neurodegenerative condition that can be divided into preclinical, prodromal, and clinical stages [1]. The prodromal stage, also known as mild cognitive impairment (MCI), represents a transitional phase where cognitive symptoms begin to manifest, leading to dementia [2]. Studies have suggested that the presence of other diseases (i.e. comorbidities) could affect the evolution of MCI/AD [3, 4]. Mood disorders, such as anxiety and depression, can occur decades before the onset of MCI or AD [3, 5, 6]. A previous study identified a strong positive anxiety-AD association (Hazard Ratio (HR) 3.90 [95% confidence interval 1.59–9.60]) [7]. In addition, another study found that depression was positively associated with AD (Odds Ratio (OR) 1.70 [1.00-2.70]) [8]. Furthermore, a large prospective study revealed an increased risk of progression from normal cognition to MCI in people living with depression (RR 2.35 [1.93–3.08]) [9]. It is noteworthy that another longitudinal study reported a significant inverse association between anxiety/depression and AD (anxiety OR 0.23 [0.08–0.66]; depression OR 0.34 [0.13–0.93]) [10]. However, the authors only focused on participants referred to a memory clinic, resulting in a small sample size. This may have contributed to the contradictory results with extremely large effect sizes and wide confidence intervals [10].

Given the uncertain relationship between anxiety/depression and MCI/AD, the present study aims to investigate the correlations between these conditions using the Australian Imaging, Biomarker & Lifestyle (AIBL) study dataset through both cross-sectional (baseline data) and longitudinal analyses (follow-up data). Additionally, the study aims to determine whether sex, age, apolipoprotein E (APOE) ε4, smoking, marital status, education, and alcohol consumption act as potential effect modifiers for the targeted associations.

## Methods

The present study follows the Strengthening the Reporting of Observational Studies in Epidemiology (STROBE) statement for observational studies [11] and analyzed secondary data from the AIBL study, a longitudinal prospective cohort study launched in November 2006 [12, 13], to investigate the association between anxiety/depression and MCI/AD.

### Human Ethics and Data Collection

The AIBL study was approved by the institutional ethics committees of St. Vincent’s Health and the University of Melbourne. All participants provided written informed consent prior to the assessments, and the study complied with the Helsinki Declaration of 1975. All secondary data were de-identified to protect the privacy of participants [14]. For each participant, data of demographics, medical history, lifestyle factors, APOE ε4 carrier status, and cognitive status were collected.

### Study Population

**Fig. 1:**
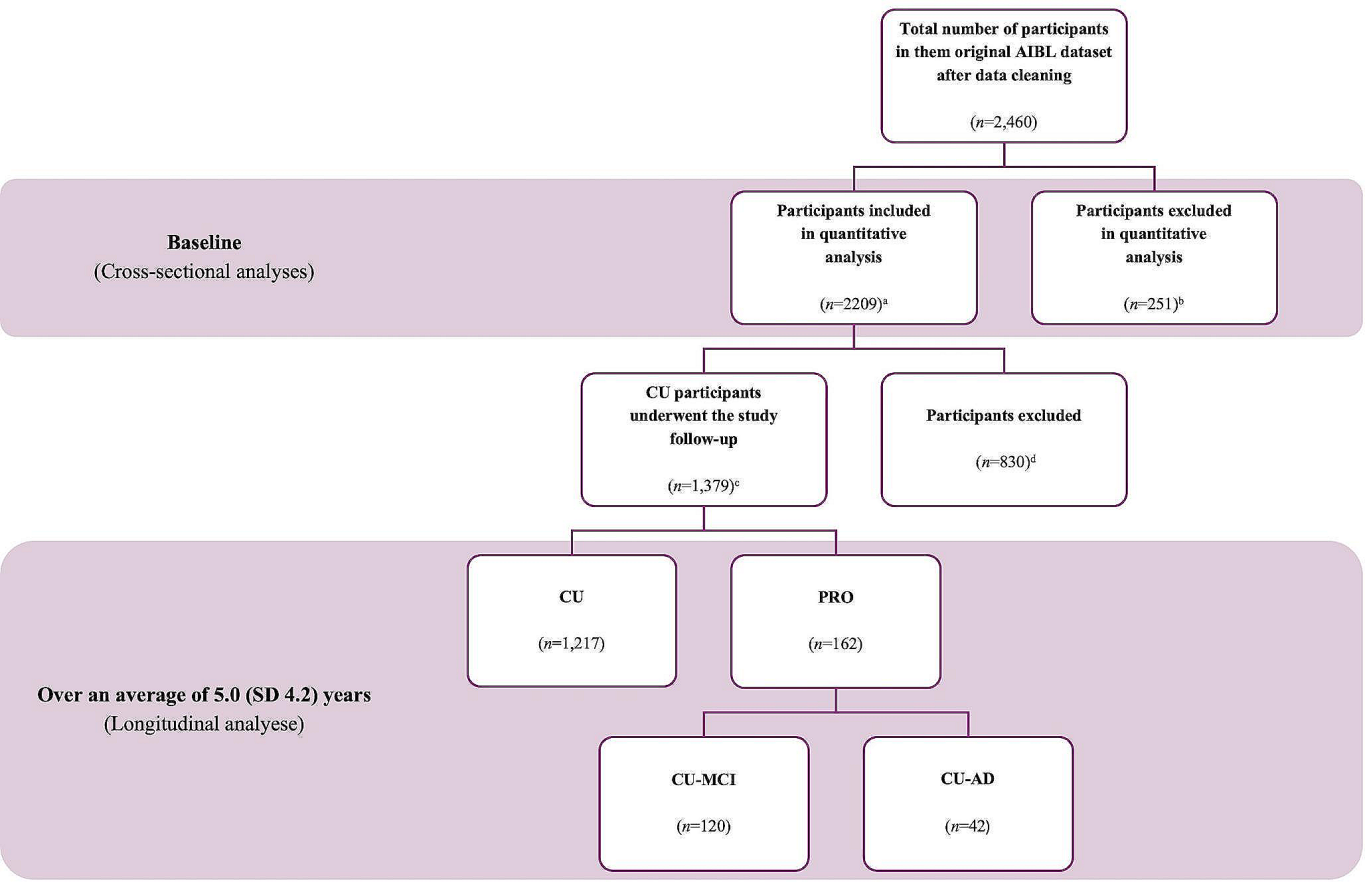
^a^At baseline, 2209 participants were included in the cross-sectional analyses (1480 CU, 358 MCI, and 371 AD). ^b^At baseline, 251 participants were excluded due to missing data for anxiety/depression and cognitive status (CU/MCI/AD). ^c^1,379 CU participants underwent the study follow-up were included in the longitudinal analyses. At the end of the study follow-up, 1,217 participants were categorized as CU, while 162 participants were categorized as experiencing cognitive decline (PRO: 120 CU-to-MCI, 42 CU-to-AD). ^d^ At the start of the study follow-up, 729 participants were excluded due to being diagnosed with MCI/AD at baseline (358 with MCI and 371 with AD). In addition, 101 participants were diagnosed with non-Alzheimer's dementia (not an outcome of interest, *n* = 29) or lost to follow-up (*n* = 72)

For cross-sectional analyses, 251 participants with missing core data were excluded, leaving 2,209 participants categorized as cognitively unimpaired (CU, *n* = 1,480), MCI (*n* = 358), or AD (*n* = 371) at baseline. For longitudinal analyses, 729 participants diagnosed with MCI/AD at baseline were excluded to ensure that the cohort was initially free of cognitive impairment. In addition, 29 were excluded due to being diagnosed with non-Alzheimer’s dementia, and 72 were excluded for lost to follow-up. Over an average follow-up of 5.0 years (SD 4.2), 1,217 participants remained CU, while 162 participants developed MCI/AD (refer to as PRO group). Detailed information is shown in Fig. 1.

### Exposures and Outcomes

In the AIBL study, information on exposure (anxiety, depression) was collected through self-report [12, 13]. Participants were asked whether they currently have or have a history of anxiety or depression. The participants were asked (1) do you have a history of anxiety or depression; (2) when you were diagnosed; (3) what kind of anxiety or depression, its severity, and the treatment you received. Those live with anxiety or depression were referred to as A + or D+, respectively, while those without were referred to as A- or D-. It needs to be noted that anxiety and depression can occur concurrently, and participants with neither anxiety nor depression were grouped as MD-, and the remaining participants were grouped as MD+. In the present study, MCI, AD, and PRO (CU-to-MCI/AD) were the outcomes of interest. Baseline cognitive statuses (CU/MCI/AD) and cognitive changes throughout the study follow-up were determined as previously described [12, 13].

### Covariates

Sex, age, APOE ε4 status, smoking, marital status, education, and alcohol consumption were selected as covariates for the target associations given their potential impact on the evolution of MCI/AD [15–21]. Information on these covariates was obtained from self-report/questionnaires and APOE genetic test at the study baseline. Sex was categorized as ‘male’ and ‘female’. Age was considered as a continuous variable in years. APOE ε4 carrier status was categorized as carrier “Yes” and non-carrier “No”. Categorization for other covariates are “non-smoker”, “former smoker” and “current smoker” for smoking; “single”, “couple” and “status change” (including widowed and divorced) for marital status; “7–8 years”, “9–12 years”, “13–15 years” and “15+ years” for education; “non-drinker”, “light drinker” (alcohol consumption < 3 days per week), “moderate drinker” (alcohol consumption 3–6 days per week) and “heavy drinker” (4 or more standard drinks per day for males and 2 or more standard drinks per day for females) for alcohol consumption.

### Data Analysis

Data analysis was conducted using Stata statistical software (version 17.0; College Station, TX) [22]. ORs were employed to measure the target associations for both cross-sectional and longitudinal analyses. Univariable and multivariable logistic regression models (LRMs) were used to determine the crude and adjusted ORs, respectively. We applied a standard adjustment model (referred to as M1), which controlled for seven covariates (sex, age, APOE ε4 status, smoking, marital status, education, and alcohol consumption). Due to the frequent co-occurrence of anxiety and depression, an advanced adjustment model (referred to as M2) was also used to adjust for each of these two mood disorders after controlling for the seven covariates, where applicable. Furthermore, we examined effect modification by each covariate using multivariable LRMs with interaction terms, and likelihood ratio tests (LRTs) were conducted to assess whether these covariates had significant modifying effects on the target associations. Statistical differences were assessed using an ANOVA test for quantitative variables (age in years) and a Chi-Square test for qualitative variables (sex, APOE ε4 status, smoking, marital status, education level, and alcohol consumption). Post-hoc tests were performed where required. All statistical tests were two-sided, and 95% confidence intervals (CIs) were reported for each calculated OR and LRT to assess uncertainty. Statistical significance was defined as *p* < 0.05.

#### Cross-sectional and Longitudinal Analyses

For cross-sectional analyses, baseline cognitive status (CU, MCI, and AD) and baseline mood disorder status (A+/-, D+/-, or MD+/-) were used to investigate the associations between these conditions. Additionally, in the longitudinal analyses, we examined the association between baseline mood disorder status and changes in cognitive status (from baseline to the last follow-up point) to understand how mood disorders may impact cognitive impairment progression over time.

#### Sensitivity Analysis

Sensitivity analysis was performed to assess the association between anxiety/depression and CU-to-MCI/AD transitions, aiming to evaluate whether reverse causality influenced the observed link found in the cross-sectional analyses.

## Results

### Participants Description

Group characteristics and comparisons (*n* = 2,209) are presented in Table 1A. There were no missing data for age and sex. For alcohol consumption, data for 14.2% of participants were missing, while the other covariates had less than 10% missing values. In this cohort, the overall mean age was 72.3 years (standard deviation, SD 7.4), and there were more females than males (55.4% vs. 44.6%). A significantly lower proportion of CU (28.1%) participants carry the APOE ε4 allele compared to MCI (51.6%, *p* < 0.001) and AD (63.7%, *p* < 0.001) participants. A significantly lower proportion of CU (33.7%) participants were former smokers compared to MCI (43.9%, *p* < 0.001) and AD (42.5%, *p* = 0.002) participants. A significantly higher proportion of AD participants were coupled compared to CU participants (72.5% vs. 65.9%, *p* = 0.016). A significantly higher proportion of MCI participants completed 9–12 years of education compared to CU participants (46.9% vs. 35.5%, *p* < 0.001). About 20% of AD participants completed 7–8 years of education, while it was a lower proportion for CU participants (18.3% vs. 6.6%, *p* < 0.001). Most CU participants (46.3%) were moderate drinkers compared to MCI (31.8%, *p* < 0.001) and AD (24.3%, *p* < 0.001) participants. For each cognitive status group, about a quarter of the participants were heavy drinkers. A total of 383 (17.3%) participants reported anxiety, and 446 (20.2%) reported depression at the study baseline.

The baseline characteristics of follow-up participants (*n* = 1,379) are shown in Table 1B. There were no missing values for age and sex, but some values were missing for smoking (10.9%) and alcohol consumption (15.2%). The other covariates contained less than 3% missing values. In this cohort, the overall mean age was 71.2 years (SD 6.6), and the mean follow-up period was 5.0 years (SD 4.2). In comparison to CU participants, the PRO group had a higher proportion of males (53.1% vs. 42.5%, *p* = 0.011). Approximately a quarter of the CU group were APOE ε4 allele carriers, while nearly half of the PRO group were APOE ε4 allele carriers (42.0% vs. 25.8%, *p* < 0.001). For both CU and PRO groups, most participants were non-smokers, with less than 4% being current smokers. A significantly higher proportion of PRO participants had a change in marital status compared to CU participants (12.4% vs. 6.5%, *p* = 0.007). In addition, a significantly higher proportion of PRO participants completed 7–8 years of education compared to CU participants (13.0% vs. 5.9%, *p* < 0.001). About 1 in 4 PRO participants lived with anxiety (27.2%) or depression (25.9%). Baseline demographic characteristics of CU-to-MCI and CU-to-AD groups can be found in the Supplementary Material (Table **S1**

### Cross-sectional Analyses

#### Association of Anxiety and Depression with MCI and AD

Cross-sectional analyses (Table 2) revealed a significant positive association between anxiety and MCI/AD, whether using the crude model or adjusting for covariates in M1/M2 models. It is noteworthy that the strength of both associations decreased after adjustment for depression (M2 model). The odds of being MCI (1.79 [1.24–2.59], *p* = 0.002) or AD (2.13 [1.44–3.15], *p* < 0.001) were higher in D + participants compared with D- participants after M1 adjustment. However, after anxiety was adjusted, the depression-MCI association became statistically nonsignificant, while depression remained significantly associated with AD (1.73 [1.12–2.69], *p* = 0.014). The strength for the depression-MCI/AD association decreased after adjusting for anxiety. When anxiety and depression were analysed as a cluster (i.e. MD), it was associated with higher odds of both MCI (1.75 [1.25–2.45], *p* = 0.001) and AD (1.82 [1.27–2.61], *p* = 0.001), after controlling for covariates.

**Table 1:** **A**: Baseline demographic characteristics of the participants

	CU (*n* = 1,480)	MCI (*n* = 358)	AD (*n* = 371)	All (*n* = 2,209)	*p*-value	Post-hoc
Covariates						
Age (years), mean (SD)	71.0 (6.6)	74.3 (7.7)	75.7 (8.8)	72.3 (7.4)	< 0.001*	a; b
Sex, number (%)					0.020*	a
Female	847 (57.2)	176 (49.2)	201 (54.2)	1,224 (55.4)		
Male	633 (42.8)	182 (50.8)	170 (45.8)	985 (44.6)		
APOE ε4 carrier status, number (%)					< 0.001*	a; b
No	1,037 (71.9)	139 (48.4)	109 (36.3)	1,285 (63.3)		
Yes	405 (28.1)	148 (51.6)	191 (63.7)	744 (36.7)		
Smoking, number (%)					0.001*	a; b
Never	839 (63.2)	170 (53.0)	197 (56.1)	1,206 (60.3)		
Former	447 (33.7)	141 (43.9)	149 (42.5)	737 (36.9)		
Current	41 (3.1)	10 (3.1)	5 (1.4)	56 (2.8)		
Marital status, number (%)					0.001*	b
Single	384 (26.0)	92 (26.0)	92 (25.1)	568 (25.9)		
Couple	972 (65.9)	244 (68.9)	266 (72.5)	1,482 (67.5)		
Status change	120 (8.1)	18 (5.1)	9 (2.5)	147 (6.7)		
Education, number (%)					< 0.001*	a; b
7–8 years	98 (6.6)	45 (12.7)	66 (18.3)	209 (9.5)		
9–12 years	524 (35.5)	166 (46.9)	142 (39.4)	832 (38.0)		
13–15 years	311 (21.1)	63 (17.8)	74 (20.6)	448 (20.5)		
15+	544 (36.8)	80 (22.6)	78 (21.7)	702 (32.0)		
Alcohol consumption, number (%)					< 0.001*	a; b
Non-drinker	183 (14.5)	63 (20.9)	105 (31.9)	351 (18.5)		
Light drinker	237 (18.8)	55 (18.2)	52 (15.8)	344 (18.2)		
Moderate drinker	585 (46.3)	96 (31.8)	80 (24.3)	761 (40.2)		
Heavy drinker	259 (20.5)	88 (29.1)	92 (28.0)	439 (23.2)		
Mood disorders						
Anxiety, number (%)					< 0.001*	a; b
A- (No)	1,274 (86.1)	281 (78.5)	271 (73.1)	1,826 (82.7)		
A+ (Yes)	206 (13.9)	77 (21.5)	100 (27.0)	383 (17.3)		
Depression, number (%)					< 0.001*	a; b
D- (No)	1,225 (82.8)	277 (77.4)	261 (70.4)	1,763 (79.8)		
D+ (Yes)	255 (17.2)	81 (22.6)	110 (29.6)	446 (20.2)		
Anxiety ± depression (MD), number (%)					< 0.001*	a; b
MD- (No)	1,130 (76.4)	246 (68.7)	230 (62.0)	1,606 (72.7)		
MD+ (Yes)	350 (23.7)	112 (31.3)	141 (38.0)	603 (27.3)		

Total participants *n* = 2,209. Differences between groups for categorical variables were tested by chi-square analyses and differences between groups for continuous variables were tested by analysis of variance. The data are presented as mean (standard deviation, SD) or number (%). *p-value is statistically significant (*p* < 0.05). Post-hoc test [*p* < 0.05]: (a) MCI vs. CU; (b) AD vs. CU.

**Table 1:** **B**: Baseline demographic characteristics of PRO participants

	CU (*n* = 1,217)	PRO (*n* = 162)	All (*n* = 1,379)	*p*-value
**Covariates**				
Age (years), mean (SD)	70.9 (6.5)	73.0 (7.0)	71.2 (6.6)	0.216
Sex, number (%)				0.011*
Female	700 (57.5)	76 (46.9)	776 (56.3)	
Male	517 (42.5)	86 (53.1)	603 (43.7)	
APOE ε4 carrier status, number (%)				< 0.001*
ε4/no	875 (74.2)	94 (58.0)	969 (72.3)	
ε4/Yes	304 (25.8)	68 (42.0)	372 (27.7)	
Smoking, number (%)				0.473
Never	688 (63.9)	93 (60.8)	781 (63.6)	
Former	355 (33.0)	57 (37.2)	412 (33.5)	
Current	33 (3.1)	3 (2.0)	36 (2.9)	
Marital status, number (%)				0.022*
Single	317 (26.1)	43 (26.5)	360 (26.2)	
Couple	817 (67.4)	99 (61.1)	916 (66.6)	
Status change	79 (6.5)	20 (12.4)	99 (7.2)	
Education, number (%)				0.001*
7–8 years	71 (5.9)	21 (13.0)	92 (6.7)	
9–12 years	411 (33.9)	66 (40.7)	477 (34.7)	
13–15 years	266 (21.9)	28 (17.3)	294 (21.3)	
15+	466 (38.4)	47 (29.0)	513 (37.3)	
Alcohol consumption, number (%)				0.244
Non-drinker	139 (13.6)	26 (17.7)	165 (14.1)	
Light drinker	201 (19.7)	20 (13.6)	221 (18.9)	
Moderate drinker	476 (46.5)	69 (46.9)	545 (46.6)	
Heavy drinker	207 (20.2)	32 (21.8)	239 (20.4)	
**Mood disorders**				
Anxiety, number (%)				0.012*
A- (No)	988 (81.2)	118 (72.8)	1,106 (80.2)	
A+ (Yes)	229 (18.8)	44 (27.2)	273 (19.8)	
Depression, number (%)				0.121
D- (No)	966 (79.4)	120 (74.1)	1,086 (78.8)	
D+ (Yes)	251 (20.6)	42 (25.9)	293 (21.2)	
Anxiety ± depression (MD), number (%)				0.053
MD- (No)	871 (71.6)	104 (64.2)	975 (70.7)	
MD+ (Yes)	346 (28.4)	58 (35.8)	404 (29.3)	

Total participants *n* = 1,480. Differences between groups for categorical variables were tested by chi-square analyses and differences between groups for continuous variables were tested by analysis of variance. The data are presented as mean (standard deviation, SD) or number (%). * *p*-value is statistically significant (*p* < 0.05)

**Table 2:** Odds of incident MCI and AD in A+, D + or MD + individuals compared with A-, D- or MD- individuals as the reference

Odds Ratio (OR) [95% CI], *p*-value		
Mood disorder	MCI	AD
**Anxiety**	**Reference (A-)**	
Crude	1.69 [1.27–2.27], *p* < 0.001*	2.28 [1.73-3.00], *p* < 0.001*
M1^†^	1.92 [1.30–2.82], *p* = 0.001*	2.11 [1.40–3.18], *p* < 0.001*
M2^‡^	1.62 [1.06–2.48], *p* = 0.026*	1.65 [1.04–2.60], *p* = 0.033*
**Depression**	**Reference (D-)**	
Crude	1.40 [1.06–1.86], *p* = 0.018*	2.02 [1.56–2.63], *p* < 0.001*
M1^†^	1.79 [1.24–2.59], *p* = 0.002*	2.13 [1.44–3.15], *p* < 0.001*
M2^‡^	1.49 [0.99–2.23], *p* = 0.055	1.73 [1.12–2.69], *p =* 0.014*
**Anxiety ± depression (MD)**	**Reference (MD-)**	
Crude	1.47 [1.14–1.89], *p* = 0.003*	1.98 [1.55–2.52], *p* < 0.001*
M1^†^	1.75 [1.25–2.45], *p* = 0.001*	1.82 [1.27–2.61], *p =* 0.001*

* *p*-value is statistically significant (*p* < 0.05)

^†^ Standard Adjustment Model (M1): Controlled for seven covariates (age, sex, APOE ε4 carrier status, smoking status, marital status, education level and alcohol consumption) only.

^‡^ Advanced Adjustment Model (M2): Adjusted for each of these two mood disorders (anxiety/depression) after controlling for the seven covariates. It is noteworthy that M2 adjustment was not conducted for the associations between MD and MCI/AD in the present study.

#### Effect Modification by each Covariate on the Association of Anxiety/Depression with MCI/AD

The likelihood ratio test (LRT) was employed to evaluate whether the target associations were effect-modified by each selected covariate (Table 3, upper panel). Results indicated no significant effect modification for the anxiety/depression-MCI/AD association across any of the selected covariates. There was moderate evidence (LRT *p* = 0.046) against the null hypothesis that the association between MD and MCI was not modified by age. In addition, there was moderate to strong evidence (LRT *p* = 0.024) against the null hypothesis that the association between MD and AD was not modified by sex.

**Table 3:** P-values for likelihood ratio tests (LRTs) on the association of anxiety, depression and MD with MCI and AD when assessing effect modification by each covariate, and stratified odds ratios for the target associations

Likelihood ratio test (LRT) *p*-value				
Mood disorder	MCI		AD	
	M1	M2	M1	M2
**Anxiety**				
Age (1-year increment in age)	0.109	0.116	0.110	0.094
Sex (reference: female)	0.538	0.643	0.091	0.141
APOE ε4 carrier status (reference: ε4/no)	0.147	0.137	0.641	0.608
Smoking status (reference: non-smoker)	0.085	0.071	*N/A*^†^	*N/A*^†^
Marital status (reference: single)	0.500	0.518	0.180	0.156
Education level (reference: 7–8 years)	0.063	0.094	0.657	0.720
Alcohol consumption (reference: non-drinker)	0.335	0.320	0.637	0.568
**Depression**				
Age (1-year increment in age)	0.220	0.244	0.480	0.432
Sex (reference: female)	0.328	0.350	0.077	0.091
APOE ε4 carrier status (reference: ε4/no)	0.866	0.879	0.821	0.816
Smoking status (reference: non-smoker)	0.646	0.668	*N/A*^†^	*N/A*^†^
Marital status (reference: single)	0.849	0.805	0.337	0.315
Education level (reference: 7–8 years)	0.640	0.678	0.508	0.415
Alcohol consumption (reference: non-drinker)	0.441	0.354	0.449	0.396
**Anxiety ± depression (MD)**	**M1**		**M1**	
Age (1-year increment in age)	0.046*		0.118	
Sex (reference: female)	0.157		0.024*	
APOE ε4 carrier status (reference: ε4/no)	0.237		0.513	
Smoking status (reference: non-smoker)	0.905		*N/A*^†^	
Marital status (reference: single)	0.562		0.117	
Education level (reference: 7–8 years)	0.175		0.999	
Alcohol consumption (reference: non-drinker)	0.309		0.521	
**Effect modifier**	**Odd ratio (OR) [95% CI]**, ***p*-value**			
**Age**	**MD-MCI (LRT*p*** **= 0.046)**			
1-year increment in age	1.04 [1.01–1.08], *p* = 0.033*			
**Sex**	**MD-AD (LRT*p*** **= 0.024)**			
Female	1.31 [0.83–2.08], *p* = 0.215			
Male	3.04 [1.72–5.37], *p* < 0.001*			

The upper panel of this table shows the *p*-values for LRT tests, while the lower panel displays the ORs for the association between MD and MCI, stratified by age, and the ORs for the association between MD and AD stratified by sex.

* *p*-value is statistically significant (*p* < 0.05)

^†^ The *p*-value cannot be obtained due to the zero number of subjects in subgroups, resulting in no valid statistics for certain stratified associations.

The MD-MCI/AD associations, stratified by age and sex, are summarized in Table 3**(**lower panel). For every 1-year increase in age, MD + participants showed higher odds of MCI (1.04 [1.01–1.08], *p* = 0.033) compared to MD- participants after M1 adjustment. Concerning sex, female MD + participants had higher odds of developing AD (1.31 [0.83–2.08]) than female MD- participants after controlling for M1 covariates. However, there was only weak evidence against the null hypothesis that there is no association between MD and AD in females (*p* = 0.215). In addition, male MD + participants exhibited a greater likelihood of being AD (3.04 [1.72–5.37], *p* < 0.001) than male MD- participants after controlling M1 covariates.

### Longitudinal Analyses

#### Association of Anxiety/Depression with PRO

In longitudinal analyses (Table 4), a significant positive association between anxiety and PRO was observed after controlling for M1covariates (1.56 [1.02–2.38], *p* = 0.041). However, this association was no longer statistically significant after adjusting for depression. Regardless of whether the crude model or M1/M2 adjustment was used, a positive but statistically nonsignificant depression-PRO association was observed, as indicated by the corresponding confidence intervals crossing the null value, reflecting uncertainty in the association. Notably, the strength of anxiety/depression-PRO associations decreased after M2 adjustment. A significant positive MD-PRO association (1.48 [1.01–2.20], *p* = 0.047) was observed after adjusting for M1 covariates.

**Table 4:** Odds of incident PRO in A+, D + or MD + individuals compared with A-, D- or MD- individuals as the reference

Odds Ratio (OR) [95% CI], *p*-value	
Mood disorder	PRO
**Anxiety**	Reference (A-)
Crude	1.61 [1.11–2.34], *p* = 0.013*
M1^†^	1.56 [1.03–2.39], *p* = 0.041*
M2^‡^	1.45 [0.88–2.36], *p* = 0.141
**Depression**	Reference (D-)
Crude	1.34 [0.92–1.97], *p* = 0.122
M1^†^	1.40 [0.91–2.13], *p* = 0.123
M2^‡^	1.17 [0.72–1.91], *p* = 0.530
**Anxiety ± depression (MD)**	Reference (MD-)
Crude	1.40 [0.99–1.98], *p* = 0.054
M1^†^	1.48 [1.01–2.20], *p* = 0.047*

* *p*-value is statistically significant (*p* < 0.05)

^†^ Standard Adjustment Model (M1): Controlled for seven covariates (age, sex, APOE ε4 carrier status, smoking status, marital status, education level and alcohol consumption) only

^‡^ Advanced Adjustment Model (M2): Adjusted for each of these two mood disorders (anxiety/depression) after controlling for the seven covariates. It is noteworthy that M2 adjustment was not conducted for the associations between MD and PRO in the present study

#### Effect Modification by each Covariate on the Association of Anxiety/Depression with PRO

The LRT tests were also employed in longitudinal analyses to determine whether the target associations were effect-modified by each selected covariate. As depicted in Table 5 (upper panel), there was strong evidence against the null hypothesis that the anxiety/depression/MD-PRO associations were not modified by marital status (LRT *p* < 0.05).

**Table 5:** P-values for likelihood ratio tests (LRTs) on the association of anxiety, depression and MD with PRO when assessing effect modification by each covariate, and stratified odds ratios for the target associations

Likelihood ratio test (LRT) *p*-value		
Mood disorder	PRO	
	M1	M2
**Anxiety**		
Age (1-year increment in age)	0.575	0.547
Sex (reference: female)	0.134	0.119
APOE ε4 carrier status (reference: ε4/no)	0.050	0.051
Smoking status (reference: non-smoker)	0.334	0.339
Marital status (reference: single)	0.016*	0.015*
Education level (reference: 7–8 years)	0.209	0.201
Alcohol consumption (reference: non-drinker)	0.316	0.306
**Depression**		
Age (1-year increment in age)	0.207	0.172
Sex (reference: female)	0.350	0.382
APOE ε4 carrier status (reference: ε4/no)	0.396	0.453
Smoking status (reference: non-smoker)	0.867	0.833
Marital status (reference: single)	0.005*	0.004*
Education level (reference: 7–8 years)	0.226	0.224
Alcohol consumption (reference: non-drinker)	0.863	0.789
**MD**		
Age (1-year increment in age)	0.736	
Sex (reference: female)	0.525	
APOE ε4 carrier status (reference: ε4/no)	0.203	
Smoking status (reference: non-smoker)	0.608	
Marital status (reference: single)	0.002*	
Education level (reference: 7–8 years)	0.286	
Alcohol consumption (reference: non-drinker)	0.507	
**Effect modifier**	**Odd ratio (OR) [95% CI]**, ***p*-value**	
**Marital status**	**Anxiety-PRO**	
	**M1 (LRT*p*** **= 0.016)**	**M2 (LRT*p*** **= 0.015)**
Single	2.88 [1.36–6.10], *p* = 0.006*	2.67 [1.22–5.86], *p* = 0.014*
Couple	1.52 [0.87–2.65], *p* = 0.138	1.40 [0.76–2.58], *p* = 0.279
Status change	0.28 [0.06–1.37], *p* = 0.116	0.25 [0.05–1.29], *p* = 0.099
	**Depression-PRO**	
**Marital status**	**M1 (LRT*p*** **= 0.005)**	**M2 (LRT*p*** **= 0.004)**
Single	3.26 [1.56–6.78], *p* = 0.002*	2.72 [1.27–5.85], *p* = 0.010*
Couple	1.11 [0.63–1.96], *p* = 0.720	0.91 [0.48–1.69], *p* = 0.757
Status change	0.19 [0.02–1.60], *p* = 0.127	0.14 [0.02–1.27], *p* = 0.081
**Marital status**	**MD-PRO (LRT*p*** **= 0.002)**	
Single	3.22 [1.55–6.68], *p* = 0.002*	
Couple	1.37 [0.83–2.26], *p* = 0.222	
Status change	0.22 [0.04–1.06], *p* = 0.060	

The upper panel of this table shows the *p*-values for LRT tests, while the lower panel displays the ORs for the association between MD/anxiety/depression and PRO, stratified by marital status

* *p*-value is statistically significant (*p* < 0.05)

The anxiety/depression/MD-PRO associations stratified by marital status are summarized in Table 5 (lower panel). Single A + participant had higher odds of PRO than single A- participant after adjustment (M1: 2.88 [1.36–6.10], *p* = 0.006; M2: 2.67 [1.22–5.86], *p* = 0.014). The odds of PRO were higher in single D + participants compared with single D- participants after adjustment (M1: 3.26 [1.56–6.78], *p* = 0.002; M2: 2.72 [1.27–5.85], *p* = 0.010). However, nonsignificant associations were observed in both couple and status change subgroups. Interestingly, substantial negative anxiety/depression-PRO links were found in status change group (ORs ranging from 0.14 to 0.28) after M1 or M2 adjustment, although without statistical significance (*p* > 0.05). Furthermore, the odds of PRO were higher in single MD + participants compared with single MD- participants after controlling for selected covariates (3.22 [1.55–6.68], *p* = 0.002).

### Sensitivity Analyses for Association of Anxiety, Depression, and MD with CU-MCI/AD

As depicted in **Supplementary material (Table S2)**, no significant association between anxiety/depression/MD and CU-MCI was observed in the sensitivity analyses. For CU-AD, we observed that anxiety was significantly positively associated with CU-AD (anxiety: 2.73 [1.26–5.87], *p* = 0.010) after M1 adjustment but not for depression. Similar to the main analyses mentioned before, the strength of these associations decreased after M2 adjustment. Additionally, MD was associated with higher odds of CU-AD (2.18 [1.06–4.47], *p* = 0.034) after M1 adjustment.

## Discussion

We conducted an exploratory study that demonstrated a positive cross-sectional and longitudinal association between anxiety/depression and MCI/AD. Age, sex, and marital status were identified as three effect modifiers for the corresponding associations. Although it remains uncertain whether anxiety and depression symptoms are manifestations of the underlying dementia process or themselves induce neurochemical changes that promote the neuropathological changes of dementia, our study can provide important epidemiological evidence to inform dementia prevention and surveillance.

The cross-sectional analyses based on baseline data from 2,209 participants showed that anxiety and depression were significantly associated with MCI or AD, regardless of whether crude or M1 adjustment analyses were used. However, our findings are at variance with a recent United States study (anxiety-MCI: HR 1.31 [0.48–3.57]; depression-MCI: HR 1.47 [0.68–3.17]) [23]. The lack of association in that study could be attributed to a relative low sample size (anxiety-MCI: *n* = 50; depression-MCI: *n* = 58), potentially limiting the statistical power, as indicated by wider confidence intervals [23]. In relation to the anxiety/depression-AD association, our findings are consistent with two data analysis studies conducted in Spain (HR 3.20 [1.39–7.40]) and South Korea (HR 4.41 [4.04–4.81]) [7, 24, 25]. However, their reported associations are stronger than that observed in AIBL, possibly due to differences in study setting, methodology and geography. Interestingly, the cross-sectional depression-MCI association became insignificant after M2 model adjustment in the present study, suggesting that it was possibly overestimated without further control for anxiety. This underscores the importance of considering both anxiety and depression simultaneously when assessing their associations with cognitive outcomes, which has not been performed in several prior studies [, 24, 26].

The MD-MCI association was found to be effect-modified by age (LRT *p* = 0.046). Stratified OR revealed 1.04-fold higher odds of MCI in MD + participants compared with MD- participants for every 1-year increment in age. The MD-AD association was found to be effect-modified by sex (LRT *p* = 0.024). Stratified OR revealed that MD was more strongly associated with cognitive impairment in males than in females. Our results are in accordance with other studies; for example, Fuhrer et al. [27] and Dal Forno et al. [28] found that depressive symptoms significantly increased the risk of AD in males but not in females, and Nguyen et al. found a higher odds ratio in males with anxiety than females with anxiety [4]. The disproportional increase in the risk of MCI in MD + versus MD- participants and the gender effect seen in MD-AD association warrants further investigation. Regardless, males with mood disorders can possibly be identified as a high-risk population, to whomclinicians could offer dementia prevention and surveillance programs. Interestingly, although the distribution of MCI (*n* = 358) and AD (*n* = 371) was almost balanced for the current study, the strength of the associations regarding MCI was consistently lower than AD. This could be attributed to MCI having heterogenous underlying causes [29–32]. Future analyses could be performed on MCI participants with their brain levels of amyloid-beta assessed using PET imaging, to better understand the association between anxiety/depression and Aβ-positive MCI [33].

The longitudinal analyses were conducted for 1,379 participants. We observed that anxiety/MD was strongly associated with PRO after M1 adjustment. However, the anxiety-PRO association became non-significant after M2 adjustment, which suggested that the link was possibly confounded by depression. It must be noted that our sample size (A+ PRO participant *n* = 44) may not yield acceptable precision for association estimation [34]. The same sample size issue can possibly explain the nonsignificant positive depression-AD association (D+ PRO participants *n* = 42), regardless of the crude or adjusted model.

We found marital status could be considered a potential effect modifier for all the target associations. When stratified by marital status, anxiety/depression/MD was significantly positively correlated with PRO in singles but not in couples or participants with marital status change. In line with our finding, a recent study has reported that individuals who experience low mood have a heightened risk of cognitive impairment [35]. Previous studies have also reported that unmarried individuals are more likely to develop dementia [20, 36–38], which has been linked to their reduced social engagement than those married/coupled. It has been proposed that living with a partner can provide both mental and social stimulation, facilitating healthy behaviors [39]. In addition, the strength of the negative association was extremely pronounced (OR < 0.5) in those with a status change, although it did not reach statistical significance. This is consistent with a Singaporean study where a negative association between divorce and cognitive impairment was observed (OR 0.61 [0.21–1.79]) [40]. This interesting association is yet to be explored.

In our sensitivity analyses, we found no association between anxiety/depression/MD and CU-to-MCI. Anxiety/MD was associated with significantly higher odds of CU-AD, although no association between anxiety and CU-AD was identified after M2 adjustment. However, the small sample size, confounding effect of both mood disorders in corresponding associations, and elimination of the reverse causality were all possible reasons that could have induced these changes.

Our study possesses several strengths. To maximize the identification of the temporal sequence and eliminate reverse causality between target exposures and outcomes, we not only performed cross-sectional analyses but also longitudinally investigated the actual epidemiological link between anxiety/depression and MCI/AD among a cognitive impairment-free (at baseline) cohort. In addition, advanced adjustment was performed to reduce the confounding effect when examining these two mood disorders individually. Our study is not without limitations. Anxiety and depression are self-reported by the AIBL participants, which could introduce recall bias. In addition, the association between severity of anxiety/depression and MCI/AD was not examined in our study due to the data availability, which could be assessed in the future [24]. Furthermore, the AIBL cohort was predominantly Caucasian with a relatively high level of education, limiting the generalizability of the findings of the present study to populations of other ethnic backgrounds in regions outside Australia or to individuals with lower education levels.

## Conclusions

Our study, utilizing an Australian dataset, elucidates a positive cross-sectional association between anxiety/depression and MCI/AD. Longitudinal analysis further reveals that anxiety ± depression but not depression alone increases the development of MCI/AD. Notably, our cross-sectional analysis identifies sex as a significant effect modifier, with males exhibiting higher odds of developing AD. Additionally, longitudinal analysis highlights marital status as a significant effect modifier, with single individuals having the highest odds of developing MCI/AD. Our study provides valuable epidemiological insights that can inform clinical practice, guiding clinicians in offering targeted dementia prevention and surveillance programs to the at-risk populations.

## Electronic Supplementary Material

Below is the link to the electronic supplementary material.

Supplementary Material 1

## Data Availability

AIBL data is available up on formal request (https://aibl.org.au/), subject to the scientific committee and human Ethics approval.
