# Supplementary material for: Elucidating the Link Between Anxiety/Depression and Alzheimer’s Dementia in the Australian Imaging Biomarkers and Lifestyle (AIBL) Study
Source: J Epidemiol Glob Health. 2024 Jun 19;14(3):1130–41. doi: 10.1007/s44197-024-00266-w (PMC11442410; doi:10.1007/s44197-024-00266-w)
Supplement: Supplementary file 1 — Supplementary Material 1 [file 44197_2024_266_MOESM1_ESM.docx]

**SUPPLEMENTARY MATERIAL**

**Table S1. Baseline demographic characteristics of the PRO participants (CU-to-MCI/AD).**

|  | **CU-MCI (*n*=120)** | **CU-AD (*n*=42)** |
| --- | --- | --- |
| **Covariates** | | |
| **Age (years), mean (SD)** | 72.0 (7.0) | 75.8 (6.5) |
| **Sex, number (%)** |  |  |
| Female | 57 (47.5) | 19 (45.2) |
| Male | 63 (52.5) | 23 (54.8) |
| **APOE ε4 carrier status number (%)** |  |  |
| ε4/no | 74 (61.7) | 20 (47.6) |
| ε4/Yes | 46 (38.3) | 22 (52.4) |
| **Smoking status, number (%)** |  |  |
| Never | 69 (61.6) | 24 (58.5) |
| Former | 40 (35.7) | 17 (41.5) |
| Current | 3 (2.7) | / |
| **Marital status, number (%)** |  |  |
| Single | 37 (30.8) | 6 (14.3) |
| Couple | 67 (55.8) | 32 (76.2) |
| Status change | 16 (13.4) | 4 (9.5) |
| **Education, number (%)** |  |  |
| 7-8 years | 18 (15.0) | 3 (7.1) |
| 9-12 years | 51 (42.5) | 15 (35.7) |
| 13-15 years | 19 (15.8) | 9 (21.5) |
| 15+ | 32 (26.7) | 15 (35.7) |
| **Alcohol consumption, number (%)** |  |  |
| Non-drinker | 19 (17.1) | 7 (19.4) |
| Light drinker | 15 (13.5) | 5 (13.9) |
| Moderate drinker | 53 (47.8) | 16 (44.4) |
| Alcoholism | 24 (21.6) | 8 (22.3) |
| **Mood disorders** | | |
| **Anxiety, number (%)** |  |  |
| A- (No) | 90 (75.0) | 28 (66.7) |
| A+ (Yes) | 30 (25.0) | 14 (33.3) |
| **Depression, number (%)** |  |  |
| D- (No) | 91 (75.8) | 29 (69.0) |
| D+ (Yes) | 29 (24.2) | 13 (31.0) |
| **Anxiety ± depression (MD), number (%)** |  |  |
| MD- (No) | 79 (65.8) | 25 (59.5) |
| MD+ (Yes) | 41 (34.2) | 17 (40.5) |

Differences between groups for categorical variables were tested by chi-square analyses and differences between groups for continuous variables by analysis of variance. The data are presented as mean (standard deviation, SD) or number (%).

* *p*-value is statistically significant (*p*<0.05).

**Table S2: Odds of incident CU-MCI or CU-AD in A+, D+ or MD+ individuals compared with A-, D- or MD- individuals as the reference.**

| **Odds Ratio (OR) [95% CI], *p*-value** | | |
| --- | --- | --- |
| **Mood disorder** | **CU-MCI** | **CU-AD** |
| **Anxiety** | Reference (A-) | |
| Crude | 1.44 [0.93-2.23], *p*=0.104* | 2.16 [1.12-4.16], *p*=0.022* |
| M1^†^ | 1.28 [0.78-2.11], *p*=0.321* | 2.73 [1.26-5.87], *p*=0.010* |
| M2^‡^ | 1.23 [0.70-2.17], *p*=0.471* | 2.30 [0.95-5.54], *p*=0.064* |
| **Depression** | Reference (D-) | |
| Crude | 1.23 [0.79-1.91], *p*=0.364* | 1.73 [0.88-3.37], *p*=0.110* |
| M1^†^ | 1.21 [0.74-1.97], *p*=0.458* | 2.10 [0.98-4.48], *p*=0.056* |
| M2^‡^ | 1.09 [0.62-1.92], *p*=0.760* | 1.43 [0.59-3.45], *p*=0.425* |
| **MD** | Reference (MD-) | |
| Crude | 1.31 [0.88-1.94], *p*=0.187* | 1.71 [0.91-3.21], *p*=0.094* |
| M1^†^ | 1.29 [0.83-2.03], *p=*0.260* | 2.18 [1.06-4.47], *p*=0.034* |

* *p*-value is statistically significant (*p*<0.05).

^†^ Standard Adjustment Model (M1): Controlled for 7 covariates (age, sex, APOE ε4 carrier status, smoking status, marital status, education level and alcohol consumption) only.

^‡^ Advanced Adjustment Model (M2): Adjusted for each of these two mood disorders (anxiety/depression) after controlling for the 7 covariates. It is noteworthy that M2 adjustment was not conducted for the associations between MD and CU-MCI/CU-AD in the present study.
